# MiRNAs as Anti-Angiogenic Adjuvant Therapy in Cancer: Synopsis and Potential

**DOI:** 10.3389/fonc.2021.705634

**Published:** 2021-12-09

**Authors:** Behnaz Lahooti, Sagun Poudel, Constantinos M. Mikelis, George Mattheolabakis

**Affiliations:** ^1^ Department of Pharmaceutical Sciences, School of Pharmacy, Texas Tech University Health Sciences Center, Amarillo, TX, United States; ^2^ School of Basic Pharmaceutical and Toxicological Sciences, College of Pharmacy, University of Louisiana Monroe, Monroe, LA, United States; ^3^ Department of Pharmacy, University of Patras, Patras, Greece

**Keywords:** angiogenesis, adjuvant therapy, miRNAs, drugs, combinatorial

## Abstract

Angiogenesis is a key mechanism for tumor growth and metastasis and has been a therapeutic target for anti-cancer treatments. Intensive vascular growth is concomitant with the rapidly proliferating tumor cell population and tumor outgrowth. Current angiogenesis inhibitors targeting either one or a few pro-angiogenic factors or a range of downstream signaling molecules provide clinical benefit, but not without significant side effects. miRNAs are important post-transcriptional regulators of gene expression, and their dysregulation has been associated with tumor progression, metastasis, resistance, and the promotion of tumor-induced angiogenesis. In this mini-review, we provide a brief overview of the current anti-angiogenic approaches, their molecular targets, and side effects, as well as discuss existing literature on the role of miRNAs in angiogenesis. As we highlight specific miRNAs, based on their activity on endothelial or cancer cells, we discuss their potential for anti-angiogenic targeting in cancer as adjuvant therapy and the importance of angiogenesis being evaluated in such combinatorial approaches.

## Introduction

Angiogenesis is the physiological process for new blood vessel development from pre-existing ones. It is a highly coordinated, multistage process that occurs in physiological conditions, such as wound healing, the female reproductive cycle, and embryonic development, and many pathological conditions, including cancer. The angiogenic outcome highly depends on the balance of growth factors and angiogenesis inhibitors. Dysregulation of this balance leads to the increased or limited vascular network identified in a series of pathologies, such as retinopathies, inflammatory disorders, cardiovascular disorders, and tumors (1–3).

The rapid growth of tumor cells requires the continuous supply of oxygen and nutrients, the diffusion of which *in vivo* is significantly limited at 100-500 microns from the nearest capillary. Solid tumors cannot grow more than 2-3 mm in diameter and thus become dormant without vascular support (4, 5). The rapid proliferation of the tumor cells leads to their distant localization from the nearest capillary and the induction of hypoxia, a major driver of angiogenesis. Hypoxia leads to the secretion of many growth factors, such as vascular endothelial growth factor (VEGF) and basic fibroblast growth factor (bFGF), cytokines, such as interleukin 8 (IL-8), and other pro-angiogenic mediators, such as sphingosine-1 phosphate (S1P), leading to the proliferation, migration and tumor-like formation of the nearby endothelial cells (5–8). The newly formed tumor vessels are markedly distinct from the normal capillaries due to their chaotic structure characterized by the absence of proper orientation, the limited pericyte and smooth muscle cell coverage, blunt capillary ends, increased leakiness, and limited perfusion. The increased leakiness provides fertile ground for tumor cell dissemination and metastasis, while the limited mural support often leads to their collapse due to the higher interstitial pressure of the tumoral area, increasing further the hypoxic conditions (3, 5, 9).

Targeting the tumor vascular network with anti-angiogenic therapy, despite the excellent preclinical results and the high potential these provided, did not meet the expectations in the clinic, with ephemeral results and not significant benefit in overall survival in most tumors. A prominent reason for this is considered the induction of compensatory mechanisms due to increased hypoxia upon anti-angiogenic treatment, which drives the overexpression of other pro-angiogenic factors, blocks immune functionality, and limits the perfusion of cytotoxic therapies (10, 11). During the last decade, the notion of vascular normalization as an outcome of anti-angiogenic therapy has risen, which can be achieved within a short therapeutic window during anti-angiogenic therapy. Tumor vascular normalization is expected to induce the integrity of the tumor vessels providing increased mural cell support, limited leakiness, inhibition of trans-endothelial cancer cell migration and metastatic incidence, and higher perfusion, which would limit the hypoxic areas and accommodate improved anti-cancer drug delivery in the tumoral area (12–14). The majority of the studies have focused on VEGF inhibition, the main target of anti-angiogenic therapies. A precise dosage of VEGF inhibitors has been demonstrated to inhibit vascular permeability by tightening cell-to-cell contacts and recruiting pericytes. VEGF is not the sole mediator of vascular permeability, as an increasing volume of data has highlighted the involvement of other molecular players and pathways, such as Angiopoietin-2, Semaphorin 3A, nitric oxide, superoxide dismutase-3, Notch, WNT, platelet-derived growth factor-B (PDGF-B) and bone morphogenetic protein (MBP) signaling in this process (10, 11, 13, 15, 16).

Nucleic acid-based therapeutics have attracted attention for the treatment of several diseases, including cancer (17, 18), inflammation (19), or the development of vaccines, such as against SARS-CoV-2 (i.e. COVID-19) (20–22). Among the different types of nucleic acids currently under research, miRNAs, natural molecules produced by the cells frequently transcribed along with protein-expressing genes (23, 24), are commonly dysregulated in diseases, such as cancer, inflammation, and others. Not surprisingly, miRNAs were recognized as potential prognostic and diagnostic markers in cancer (23–26). More importantly, as miRNAs are small, non-coding RNAs that utilize the cell’s RNA interference mechanism to regulate multiple gene expressions, miRNAs are evaluated as therapeutic tools against cancer (23). An increasing body of literature focuses on dysregulated miRNAs for their properties as tumor suppressors or oncogenes, and on their action to either suppress or activate tumor-promoting pathways (23). Exogenous delivery of miRNA constructs, similarly to the exogenous delivery of siRNAs, aims to replace or correct observed miRNA dysregulations. Unlike siRNAs though, miRNA replacement therapies induce the expression or increase the levels of nucleic acid sequences naturally occurring in the cells, which should have an indistinguishable effect on the endogenous miRNAs (23, 24). Though this approach has limitations, the exogenous delivery of miRNAs should induce a strong beneficial effect on cells associated with the disease (i.e., cancer cells or cells of the tumor microenvironment with dysregulated miRNA expression) while having minimal effects on normal cells (i.e., absence of dysregulation) (27).

Representatively, miR-34a is characterized as a master tumor suppressor against multiple cancer types, capable of regulating proliferation, migration (28), apoptosis (29), metastasis, senescence, differentiation, and immune responses (30). Similarly, the clinical potential and translation of other miRNAs are currently undergoing. We are not outlining these studies, as several review publications focus on the current and past clinical trials [indicatively, refer to: (31–33)]. As miRNAs are expressed in all types of cells, miRNAs regulate vascular development and angiogenesis in endothelial cells (EC). Landskroner-Eiger et al. (34) summarized the importance of miRNAs in angiogenesis from the perspective of the Dicer enzyme. Dicer enzyme is a key component in the biogenesis of miRNAs, and several studies evaluated the effect of Dicer deletion/inactivation in normal vascular development. Dicer activity affected angiogenesis, attributed to defective miRNA expression, dysregulating the expression of VEGF and its receptors. As miRNA dysregulation in cancer has been well documented (35) either through cell-to-cell communication between cancer cells and EC or EC intracellular miRNA dysregulation, utilization of miRNAs as targets or regiments can benefit cancer treatments through regulation of EC function and formation of blood vessels (36, 37). There is an increasing interest in the combination of anti-angiogenic agents with traditional chemotherapeutics and several clinical trials pursued that approach (2, 38). We sought to explore the use of miRNAs for cancer treatment due to their ability to regulate angiogenesis and focus on their potential and utilization as adjuvant therapies with chemotherapeutics because of their anti-angiogenic properties. Although there is a substantial body of literature focusing on miRNAs and angiogenesis, limited work exists on their combination with chemotherapeutics predominately due to their anti-angiogenic properties. Here, we present miRNAs that are frequently studied due to their angiogenesis-inhibiting capacity and have been combined with traditional chemotherapeutics, even when the utilization of these miRNAs was not because of their anti-angiogenic properties.

## Current Anti-Angiogenic Therapies

Not long after its discovery, VEGF was characterized as a principal vascular regulator (39, 40). VEGF haploinsufficiency led to embryonic lethality due to impaired angiogenesis and blood vessel formation (41, 42). The striking impact on angiogenesis, vascular morphology, and functions upon VEGF inhibition or deficiency, along with its overexpression in most solid tumors, including lung, breast, liver, and ovarian cancers, brought it to the frontline of anti-angiogenic targets, where it remains till today. The first FDA-approved anti-angiogenic drug was bevacizumab, a monoclonal antibody against VEGF (43, 44). Bevacizumab, combined with chemotherapy, improved overall survival in colorectal cancer (45) and soon provided encouraging results when tested in ovarian, cervical, non-small cell lung cancers, and mesothelioma. Today, bevacizumab is FDA-approved for colorectal cancer, non-small cell lung cancer, renal cell carcinoma, cervical, fallopian tube cancer, peritoneal cancer, and glioblastoma, whereas it failed to provide clinical benefit in the majority of the other cancer types, including breast cancer, for which the FDA approval lasted for a short period (2). Apart from bevacizumab, other antibody-based anti-angiogenic inhibitors are ramucirumab and aflibercept, which target VEGF receptor 2 (VEGFR2) or VEGF-A, VEGF-B and placental growth factor (PlGF), respectively. The rest of the angiogenesis inhibitors include small molecule or tyrosine kinase inhibitors that target one or more signaling pathways. Some of these tyrosine kinase inhibitors, such as sunitinib and regorafenib, inhibit a wide range of molecular targets and downstream mediators. The current, clinically administered anti-angiogenic inhibitors, their molecular targets, and the approved cancer types are presented below (2, 46–54):

- Bevacizumab, targeting VEGF-A, for glioblastoma, colorectal, cervical, fallopian tube, peritoneal, non-small cell lung cancers and renal cell carcinoma.
- Ramucirumab, targeting VEGFR2, for gastric, gastroesophageal junction, non-small cell lung and colorectal cancers.
- Aflibercept, targeting VEGF-A,-B and PlGF, for colorectal cancer.
- Axitinib, targeting VEGFR1-3, for renal cell carcinoma.
- Cabozantinib, targeting VEGFR1-3, receptor tyrosine kinase (KIT), tropomyosin receptor kinase B (TRKB), anexelekto receptor tyrosine kinase (AXL), Rearranged during transfection (RET), tyrosine kinase MET, Fms-like tyrosine kinase-3 (FLT-3), TEK receptor tyrosine kinase (TIE2), for hepatocellular and renal cell carcinomas, and Medullary thyroid cancer.
- Everolimus, targeting mammalian target of rapamycin (mTOR), for breast, pancreatic, gastrointestinal, and lung cancers, Renal cell and subependymal giant cell carcinomas.
- Lenalidomide, targeting Ikaros family zinc finger protein 1,3 (IKZF1,3), E3 ubiquitin ligase, for follicular, mantle cell and marginal zone lymphomas, and multiple myeloma.
- Lenvatinib, targeting VEGFR1-3, for endometrial, hepatocellular and renal cell carcinomas and Thyroid cancer.
- Pazopanib, targeting VEGFR1-3, PDGF receptor-α/β (PDGFR-α/β), fibroblast growth factor receptor 1,2 (FGFR1,2), c-KIT, for renal cell and soft tissue carcinomas.
- Sorafenib, targeting VEGFR1-3, PDGFR-β, FLT-3, c-KIT, RAF kinases, for hepatocellular and renal cell carcinomas and thyroid cancer.
- Sunitinib, targeting VEGFR1-3, PDGFR-α/β, KIT, FLT-3, colony-stimulating factor receptor Type 1 (CSF-1R), RET, for gastrointestinal stromal and pancreatic cancers and renal cell carcinoma.
- Regorafenib, targeting VEGFR1-3, KIT, PDGFR-α/β, FGFR1,2, TIE2, discoidin domain receptor tyrosine kinase 2 (DDR2), tropomyosin receptor kinase A (TRKA), Eph2A, RAF-1, BRAF, BRAFV600E, SAPK2, PTK5, Abelson tyrosine kinase 1 (ABL), for gastrointestinal stromal and colorectal cancers and hepatocellular carcinoma.
- Thalidomide, targeting tumor necrosis factor-α (TNF-α), for multiple myeloma.
- Vandetanib, targeting VEGFR, epidermal growth factor receptor (EGFR), RET, for medullary thyroid cancer.

## Limitations and Side Effects of Anti-Angiogenic Therapies

As seen above, most anti-angiogenic drugs are targeting VEGF or VEGFR, either solely or in combination with other growth factor receptors or downstream kinases. Their administration provides encouraging clinical benefit; however, their application is not without side effects. The two most critical side effects of anti-angiogenic therapy are the induction of tumor aggressiveness along with metastatic potential and the tumor angiogenesis relapse due to the development of resistance mechanisms. The induction of tumor aggressiveness and metastatic potential upon anti-angiogenic therapy is still under debate, as it has been reported in preclinical models, but not always verified in other studies, demonstrating the variability of this phenomenon (55–57).

One of the limiting factors of anti-angiogenic therapy in cancer is that since cancer cells are not eradicated, as they do not consist the target of anti-angiogenic therapy, anti-angiogenic drugs have to be administered over long periods. The ephemeral outcome of anti-angiogenic therapy and the need for prolonged treatment eventually lead to the development of resistance upon anti-angiogenic inhibition. Resistance can be driven by the tumor cells, the stroma, immune cells, or endothelial progenitors, is mediated by the upregulation of alternative pro-angiogenic mediators, and presents cancer type- and patient-specific variability (8, 58).

Systemic anti-angiogenic drug administration, both in the case of antibody-specific VEGF inhibition and a wide range of tyrosine kinase inhibitors, can lead to organ- or tissue-specific side effects (59). A meta-analysis of five randomized clinical trials of metastatic colorectal, breast, and non-small cell lung cancers highlighted the risk of a thromboembolic event as another side effect of bevacizumab treatment in combination with chemotherapy (60). Cardiomyopathy and congestive heart failure have also been reported as side effects of anti-angiogenic inhibitors (61). Although the exact mechanism for cardiomyopathy and congestive heart failure upon VEGF signaling blockade has not yet been fully delineated, the current notion is that existing conditions depleting the vascular reserve, such as hypertension and coronary artery disease, may be considered risk factors for cardiotoxicity with VEGF signaling inhibitors, while reduced nitric oxide production, mitochondrial dysfunction and pericyte population depletion have been attributed as potential mechanisms (62, 63). It has been further preclinically demonstrated that abrogation of the physiological VEGF activity can result in increased systemic (and coronary) vascular resistance and decreased cardiac output *per se*, which is the typical reason for cardiomyopathy development. Moreover, the roles of chemotherapy or radiation therapy as concomitant factors in VEGF blockade-induced cardiotoxicity have been further reported (63, 64).

Two well-known side effects of anti-angiogenic therapy that go hand in hand are the increased rate of hemorrhage and the inhibited wound healing process, both of which are determining factors for the timing of surgical procedures (65). Pulmonary hemorrhage with fatal outcome has been reported for non-small cell lung cancer patients with different anti-angiogenesis inhibitors, such as bevacizumab, ramucirumab, sunitinib, axitinib, and motesanib. A small percentage of gastrointestinal tumor patients developed bleeding at the tumor sites, while central nervous bleeding has also been reported (61, 65). Impaired wound healing is a common issue. Angiogenesis is a pivotal part of the wound healing process, mediated by VEGF and other growth factors, thus is expected that VEGF inhibition hampers the inflammatory and granulation wound healing phases, pivotal for the wound healing process. As an alternative, milder anti-angiogenic treatments have been proposed to overcome this issue (66).To avoid wound healing deficiency of the surgical area anti-angiogenic treatment has to be terminated for at least four weeks before the surgical procedure so that the body will “wash out” the drug’s effects (61, 65).

The above demonstrate the impact and role of angiogenic factors in physiological vascular functions, the interdependence of the primary tumor and the tumor microenvironment, the need for highly targeted, vascular-specific anti-angiogenic approaches, and the consideration of anti-angiogenic therapies specifically targeting aberrant angiogenesis, without affecting regular angiogenic functions.

## MiRNA Therapeutics and Their Adjuvant Potential Against Angiogenesis

As research on miRNAs rapidly proliferates, miRNAs’ contribution in tumor suppression *via* anti-angiogenic function presented multifaceted therapeutic potentials for these molecules. miRNAs have primarily been studied for their activity as single molecules against cancer (17, 35, 67). With numerous miRNAs being able to regulate cell functions and pathways, the number of potential mechanisms of action of miRNAs in angiogenesis correlates to the potential pathways associated with angiogenesis. Nonetheless, similarly to traditional anti-angiogenic approaches, studies on miRNAs and angiogenesis have primarily focused in known, more traditional angiogenic pathways. Thus, miRNAs studies focus on angiogenic factor receptors or signaling molecules in ECs to inhibit tumor angiogenesis (68), among them more prominently being VEGF, VEGFR and PDGFR (69–71). As numerous dysregulated miRNAs have been identified in tumor samples, here, we will present a few of the miRNAs with explicit action on angiogenesis and their identified molecular targets.

miR-34a, a master tumor suppressor, is one of the best-studied miRNAs, and, hence, its activity on tumor cells and cells of the tumor microenvironment has been thoroughly evaluated. Several studies have reported on miR-34a’s ability to inhibit tumor angiogenesis. This activity takes place *via* multiple approaches, including the inhibition of the Silent Information Regulator 1 (*Sirt1*) expression, increase of the expression of acetylated Forkhead Box O1 (*FoxO1*) transcription factor, Notch1 targeting, and the *p53* protein in endothelial progenitor cells and human cancer cells (72–75). miR-34a downregulation in EC induced BCL-2-overexpression and inhibition of apoptosis, while miR-34a upregulation suppresses tumor angiogenesis, EC proliferation, migration, and tube formation (76, 77). miR-34a has also extensively been studied in combination with several chemotherapeutics, such as cisplatin (78, 79), doxorubicin (80), sorafenib (81), and paclitaxel (82), among others. Despite the well-studied anti-angiogenic properties of the miRNA, we did not find research on its combination with a chemotherapeutic agent based solely due to its anti-angiogenic properties, rather than miR-34a’s activity on the tumor cells.

Similarly, the miR-29 family, miR-29a, miR-29b, and miR-29c, are downregulated in various cancers, such as endometrial carcinoma, hepatocellular carcinoma, gastric cancer, and breast cancer (83–86). miR-29b overexpression inhibits angiogenesis and tumorigenesis *in vivo* and weakens tube formation, cell proliferation, and migration *in vitro* (83). miR-29b prevented tumor angiogenesis by targeting *AKT3* and inhibited Akt3-mediated VEGF and C-myc activations (86). In a gastric cancer mouse model, miR-29a/c prevented tumor growth, tube formation, and suppressed angiogenesis by suppressing VEGF-A expression (87). Similar to miR-34a, members of the miR-29 family have been attributed with tumor-suppressive properties and evaluated with several chemotherapeutic agents, such as cisplatin (88), and paclitaxel (89), among others. Of interest, miR-29a has been reported to contribute to doxorubicin resistance in breast cancer cells (90) and inhibit doxorubicin resistance in colon cancer cells (91). Li et al. (92) reported that cisplatin treatment induces upregulation of miR-29b, which suppressed invasion and angiogenesis of the cancer cells *in vitro* and inhibited tumor growth and neovascularization *in vivo.* The authors demonstrated that ectopic expression of miR-29b *via* intravenous administration in a subcutaneous xenograft mouse model of cervical cancer (HeLa cells) inhibited tumor growth and VEGF expression, corresponding to a decrease in vessel formation, although the authors did not evaluate this activity with the co-administration with cisplatin.

miR-221 and miR-222 modulated the angiogenic behavior of human umbilical vein endothelial cells (HUVECs) through the regulation of c-Kit expression (93). As these miRNAs were among the most abundantly expressed miRNAs in ECs (94), Nicoli et al. reported that miR-221 is essential for angiogenesis, in the zebrafish model (95). In human venous or lymphatic endothelial cells, miR-221 has been shown to inhibit angiogenesis (93, 96–98). miR-221 has been identified as oncogenic in pancreatic cancer cells (99), glioblastoma (100), breast cancer (101), and lung cancer (102), among others. miR-221/222 have also been associated with increased chemoresistance to cisplatin in ovarian (103) and breast cancer cells (104). Similar results have been reported with Adriamycin (doxorubicin) (105, 106), 5-fluorouracil (107), and paclitaxel (108). Representatively, *in vivo* analysis of downregulation of miR-221/222 through local injection in a breast cancer mouse model enhanced the cisplatin’s tumor growth inhibition capacity, but no analysis on tumor vasculature took place (104). In fact, in the *in vivo* studies of the miRNA-drug combinations, angiogenesis was not evaluated. This complex behavior is a representative example of the multi-faceted activity of miRNAs, which can be cancer- or cell-type-specific, and their combination with drugs can extend outside of the tumor cells, to the tumor microenvironment.

The expression of the most potent angiogenesis modulators in different tumors in terms of downstream targets of miRNAs has been extensively studied. Multiple miRNAs have been found to target VEGF since it is the most potent trigger for angiogenesis. miR-20 (109), miR-29b (110), miR-93 (111, 112), miR-126 (113, 114) target the 3’-UTR region of VEGF-A mRNA. Following, we provide representative examples of miRNAs with anti-angiogenic properties that also demonstrated anti-tumoral activity. miR-27b (115, 116) and miR-128 (69) suppress tumor progression and angiogenesis by targeting VEGF-C. miR-125b suppressed EC tube formation by inhibiting E-cadherin (117). miR-192 targets EGR1 and HOXB9, leading to anti-tumor and anti-angiogenic activity in human ovarian epithelial tumors (118). miR-200 family inhibited angiogenesis through direct and indirect mechanisms by targeting interleukin-8 (IL8) and CXCL1 secreted by the tumor endothelial and cancer cells (119). Overexpression of miR-190 inhibited EMT and angiogenesis by inactivating AKT-ERK signaling (120). miR-206 inhibited HGF-induced epithelial-mesenchymal transition (EMT) and angiogenesis in lung cancer, by suppressing Met/PI3k/Akt/mTOR signaling (121). miR-135a promoted cell apoptosis and inhibited cell proliferation, migration, invasion, and tumor angiogenesis by targeting the IGF-1 gene through the IGF-1/PI3K/Akt signaling pathway in non-small cell lung cancer (NSCLC) (122). Finally, miR-143 and miR-506, alone and in combination have been reported to affect angiogenesis, by inhibiting tube formation in HUVEC cells, while causing apoptosis to lung cancer cells (123).

**Figure 1:**
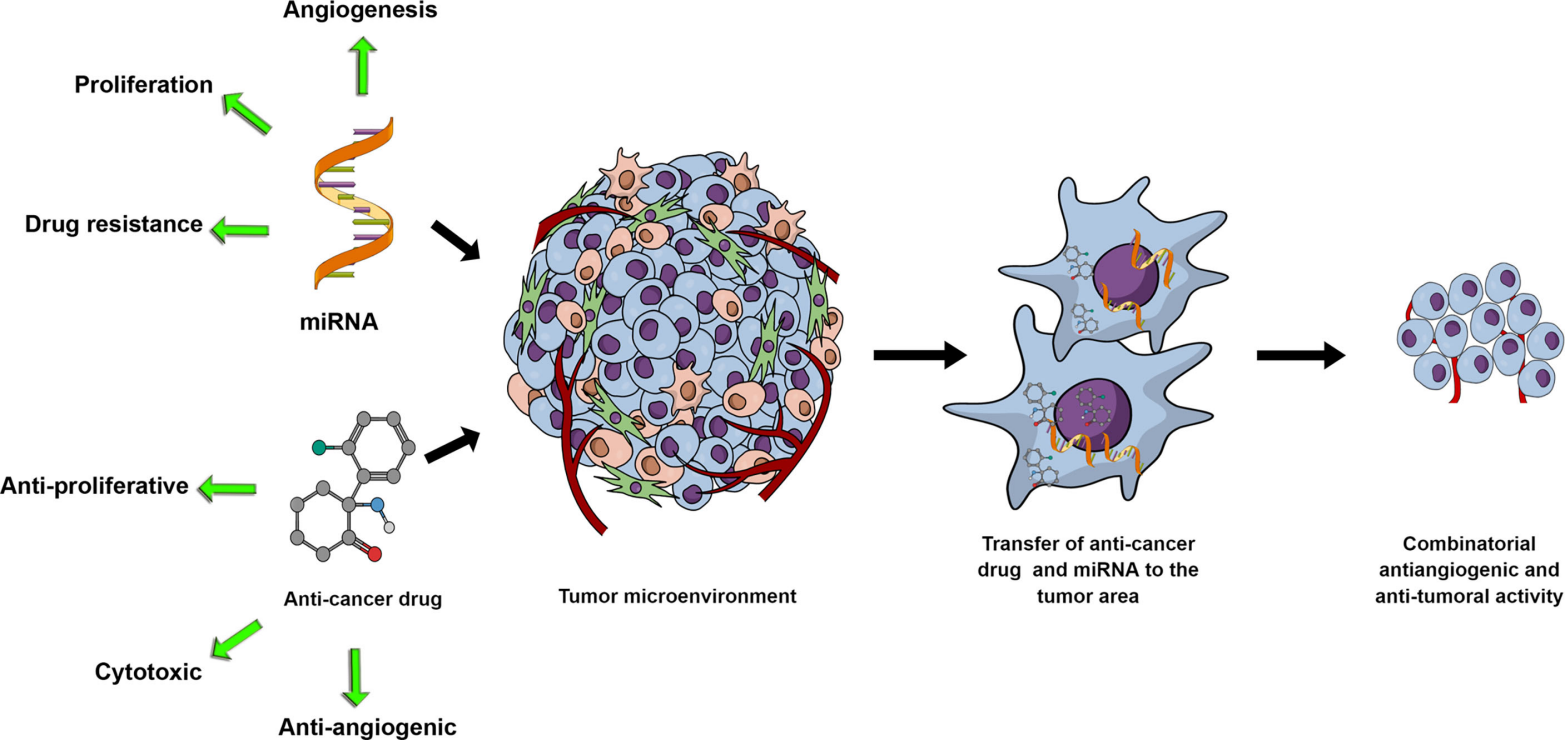
miRNA and anti-cancer drug combinations can potentially synergistically affect tumor growth through their respective activities and potential synergistic effects on the tumor cells and the tumor microenvironment. With miRNAs mediating cell proliferation, drug resistance or angiogenesis, exogenous upregulation or inhibition of miRNAs in combination with anti-proliferative, cytotoxic or anti-angiogenic drugs represents a rationally designed and promising research development.

As the VEGF family and its downregulation have been implicated in drug resistance in tumor cells (124–126), it is reasonable to predict that miRNAs with the capacity to target members of the VEGF family will become part of a cell-sensitization goal for specific chemotherapeutics. Due to this reason alone, studies of miRNA-chemotherapeutic drugs combinatorial use for cancer treatment have the potential to proliferate in the future (**Figure 1**). One representative example would be miR-126, where Zhu et al., (127) demonstrated that miR-126 decreased the minimum inhibitory concentration of Adriamycin and Vincristine by targeting VEGF-A. In **Table 1**, we present a short list of studies with miRNAs with known anti-angiogenic activity in combination with chemotherapeutics.

**Table 1:** Representative examples of combinatorial miRNA-chemotherapeutics treatments.

miRNA	Drug	Cancer	References
miR-34a	Doxorubicin	Hepatocellular carcinoma	(80)
		Osteosarcoma	(128)
	Paclitaxel	Cervical cancer	(129)
		Melanoma cancer	(130)
		Colorectal Cancer	(131)
	Docetaxel	Breast cancer	(132)
	5- Fluorouracil	Colorectal cancer	(133, 134)
Let-7c-5p	5-Flurouracil	Hepatocellular carcinoma	(135)
Anti-miR-21	Sunitinib	Glioblastoma	(136)
		Pancreatic ductal adenocarcinoma	(137)
miR-145	Sunitinib	Glioblastoma	(138)
	5-Fluorouracil	Breast cancer	(139)
miR-205	Gemcitabine	Pancreatic cancer	(140)
miR-129	5-Fluorouracil	Colorectal cancer	(141)
miR-497	5-Fluorouracil	Colorectal cancer	(142)
miR-34a and miR-27b	Docetaxel	Prostate cancer	(143)
miR-29b	Dihydroartemisinin	Cholangiocarcinoma	(144)
miR-221	Doxorubicin	Glioma	(145)
miR-192-5p	Doxorubicin	Breast cancer	(146)
miR-378a	Sorafenib	Liver cancer	(147)
miR-122, miR-338-3p	Sorafenib	Hepatocellular carcinoma	(148)
miR-193a	Taxol	Colorectal cancer	(149)
miR-143	Cisplatin	Cervical cancer	(150)
miR-29	Cisplatin	Ovarian cancer	(151)
miR-7	Doxorubicin and Temozolomide	Glioma, Cervical carcinoma, Papillary thyroid	(152)
miR-506-3p	Cisplatin	Ovarian cancer	(153)
miR-135 and miR-138	5- Fluorouracil	Colon cancer, pancreatic cancer, cervical cancer	(154)

All miRNAs listed have tumor-inhibiting properties.

Illustratively, Wang et al. (155) studied the combination of miR-30a-5p with gefitinib to overcome drug resistance *via* regulation of the insulin-like growth factor receptor-1 (IGF1R) and hepatocyte growth factor receptor signaling pathways in NSCLC both *in vitro* and *in vivo*. Liang et al. (156) formulated exosomes to simultaneously deliver the anticancer drug 5-FU and a miR-21 inhibitor oligonucleotide (miR-21i) to 5-FU-resistant colon cancer cells. This approach reversed drug resistance and significantly enhanced the drug’s cytotoxicity in 5-FU-resistant colon cancer cells, compared to the single treatment with either miR-21i or 5-FU in an *in vivo* mouse model. Similarly. miR-375-3p, which has been reported to suppress tumorigenesis and reverse chemoresistance in colon cancer, along with 5-FU co-delivered in lipid-coated calcium carbonate nanoparticles were used to study the role of miR-375-3p in 5-FU-resistance in colorectal cancer (157, 158).

## Discussion

It is evident that miRNAs can have a significant impact on angiogenesis and cancer treatment. As our knowledge on miRNA activity expands, the highly complex interaction between miRNA and angiogenesis due to autocrine or paracrine interactions will dictate the future potential of the miRNAs as therapeutic tools. One major hurdle of anti-cancer therapies, including the anti-angiogenic therapies described above, is the off-target effects due to non-specific tissue- or cell-targeting. This hurdle is further exacerbated with the miRNAs, as the tumor type and the multifaceted activity of the miRNAs can have synergistic or antagonistic therapeutic outcomes through the tumor microenvironment. Thus, the *in vivo* evaluation of the miRNAs needs to expand outside the tumor cell growth and incorporate aspects, such as angiogenesis. Another parameter to be taken into account for miRNA-based therapies is the promiscuous binding of high miRNA dose, causing multiple off-target effects. This significant hurdle of miRNA-based treatments can be resolved by miRNA cooperativity and lower miRNA doses, while it is noteworthy that the final outcome of the targets of the cooperating genes strongly depends on the cellular environment (159).

miRNA delivery has been challenging by itself, due to the nucleic acids’ rapid elimination from the circulation, the abundance of nucleases *in vivo*, and the need for a carrier for the large hydrophilic nucleic acid constructs to enter the cells (23, 24). The added complexity of the required cell type drug delivery specificity presents an additional challenge, which needs to be potentially overcome in the presence of an already impaired tumor vascular system (26). Several novel delivery carriers have been developed and studied for the delivery of miRNAs. These include micelles, polymeric nanocarriers, lipid-based carriers, viruses, inorganic carriers, and systems with long-circulating properties and/or active targeting to receptors over-expressed in cancer cells (23). Although the goal of a single organ and single-cell type targeting maybe be understandably impractical, these methodologies have provided significant benefits for minimizing off-target effects, increasing accumulation in the tumor, and preferentially increasing drug/nucleic acid concentration in specific cell types. Nonetheless, off-target effects will persist, even due to cell-to-cell communication.

This perspective has a significant impact when studying miRNA-drug combinations. Although *in vitro* analysis is fundamental to evaluate the synergistic/antagonistic behavior of a miRNA and a drug, the effect of the co-delivery of the miRNA-drug combination *in vivo* should take into consideration the anti-angiogenic properties of the miRNAs. Of course, this easily expands to other aspects of the tumor microenvironment, such as macrophages, though immunosuppressed animal models will present challenges for such evaluation.

Though we recognize that there might be published research on miRNA-drug combination focusing on angiogenesis we overlooked, it is apparent from our analysis that currently the anti-angiogenic aspect of miRNAs co-delivered with drugs is not the primary focus, or is not studied in detail or at all, even for miRNAs with known anti-angiogenic properties. Simply stated, the question arises on how much of the enhanced anti-tumoral activity of miRNA-drug combinations can be attributed to the alteration of tumor cell behavior, angiogenesis, or both. Finally, another important aspect is the toxicity potential from the miRNAs. We described above the side effects attributed to the clinically used anti-angiogenic therapies, which have a clinical history with well-defined side effects. In contrast, miRNAs have not achieved clinical translation to the same extent, and, thus, similar or other side effects may not yet have become apparent. Nonetheless, the utilization of dysregulated miRNAs, their property of being natural cell products, and the development of novel nanocarriers provide significant advantages to overcome side-effects, commonly present in traditional anti-angiogenic therapies (160). In conclusion, miRNAs are fundamentally important targets and tools for cancer therapy. They have significant potential, based alone on their multifaceted activities on the tumor cells and tumor vascular microenvironment. Identification of miRNAs with combined anti-angiogenic and anti-tumoral effects can provide significant advantages in cancer treatment, alone or in combination with clinically used chemotherapeutics.

## Author Contributions

BL, SP, CM, and GM contributed to the conception of the article, wrote and revised the final manuscript and agreed on its submission to this journal. All authors contributed to the article and approved the submitted version.

## Funding

This work was supported for GM by the College of Pharmacy, University of Louisiana Monroe start-up funding and the National Institutes of Health (NIH) through the National Institute of General Medical Science Grants 5 P20 GM103424-15, 3 P20 GM103424-15S1 and for CM in part by National Institutes of Health Grant (NCI) R15CA231339 and Texas Tech University Health Sciences Center (TTUHSC) School of Pharmacy Office of Sciences grant. The funders had no role in study design, decision to write and preparation of the manuscript.

## Conflict of Interest

The authors declare that the research was conducted in the absence of any commercial or financial relationships that could be construed as a potential conflict of interest.

## Publisher’s Note

All claims expressed in this article are solely those of the authors and do not necessarily represent those of their affiliated organizations, or those of the publisher, the editors and the reviewers. Any product that may be evaluated in this article, or claim that may be made by its manufacturer, is not guaranteed or endorsed by the publisher.

## References

[1] Sajib S Zahra FT Lionakis MS German NA Mikelis CM. Mechanisms of Angiogenesis in Microbe-Regulated Inflammatory and Neoplastic Conditions. Angiogenesis (2018) 21:1–14. doi: 10.1007/s10456-017-9583-4 29110215

[2] Zirlik K Duyster J. Anti-Angiogenics: Current Situation and Future Perspectives. Oncol Res Treat (2018) 41:166–71. doi: 10.1159/000488087 29562226

[3] Teleanu RI Chircov C Grumezescu AM Teleanu DM. Tumor Angiogenesis and Anti-Angiogenic Strategies for Cancer Treatment. J Clin Med (2019) 9:84. doi: 10.3390/jcm9010084 PMC7020037 31905724

[4] Folkman J. Tumor Angiogenesis: Therapeutic Implications. N Engl J Med (1971) 285:1182–6. doi: 10.1056/NEJM197111182852108 4938153

[5] Bielenberg DR Zetter BR. The Contribution of Angiogenesis to the Process of Metastasis. Cancer J (2015) 21:267–73. doi: 10.1097/PPO.0000000000000138 PMC4670555 26222078

[6] Ramjiawan RR Griffioen AW Duda DG. Anti-Angiogenesis for Cancer Revisited: Is There a Role for Combinations With Immunotherapy? Angiogenesis (2017) 20:185–204. doi: 10.1007/s10456-017-9552-y 28361267 PMC5439974

[7] Zahra FT Sajib MS Ichiyama Y Akwii RG Tullar PE Cobos C. Endothelial RhoA GTPase Is Essential for In Vitro Endothelial Functions But Dispensable for Physiological In Vivo Angiogenesis. Sci Rep (2019) 9:11666. doi: 10.1038/s41598-019-48053-z 31406143 PMC6690958

[8] Zahra FT Sajib MS Mikelis CM. Role of bFGF in Acquired Resistance Upon Anti-VEGF Therapy in Cancer. Cancers (2021) 13:1422. doi: 10.3390/cancers13061422 33804681 PMC8003808

[9] Schaaf MB Garg AD Agostinis P. Defining the Role of the Tumor Vasculature in Antitumor Immunity and Immunotherapy. Cell Death Dis (2018) 9:115. doi: 10.1038/s41419-017-0061-0 29371595 PMC5833710

[10] Carmeliet P Jain RK. Principles and Mechanisms of Vessel Normalization for Cancer and Other Angiogenic Diseases. Nat Rev Drug Discov (2011) 10:417–27. doi: 10.1038/nrd3455 21629292

[11] Jain RK. Antiangiogenesis Strategies Revisited: From Starving Tumors to Alleviating Hypoxia. Cancer Cell (2014) 26:605–22. doi: 10.1016/j.ccell.2014.10.006 PMC4269830 25517747

[12] Huang Y Goel S Duda DG Fukumura D Jain RK. Vascular Normalization as an Emerging Strategy to Enhance Cancer Immunotherapy. Cancer Res (2013) 73:2943–8. doi: 10.1158/0008-5472.CAN-12-4354 PMC3655127 23440426

[13] Peterson TE Kirkpatrick ND Huang Y Farrar CT Marijt KA Kloepper J. Dual Inhibition of Ang-2 and VEGF Receptors Normalizes Tumor Vasculature and Prolongs Survival in Glioblastoma by Altering Macrophages. Proc Natl Acad Sci USA (2016) 113:4470–5. doi: 10.1073/pnas.1525349113 PMC4843449 27044097

[14] Mpekris F Panagi M Voutouri C Martin JD Samuel R Takahashi S. Normalizing the Microenvironment Overcomes Vessel Compression and Resistance to Nano-Immunotherapy in Breast Cancer Lung Metastasis. Adv Sci (Weinh) (2021) 8:2001917. doi: 10.1002/advs.202001917 33552852 PMC7856901

[15] Viallard C Larrivee B. Tumor Angiogenesis and Vascular Normalization: Alternative Therapeutic Targets. Angiogenesis (2017) 20:409–26. doi: 10.1007/s10456-017-9562-9 28660302

[16] Martinez-Rey D Carmona-Rodriguez L Fernandez-Acenero MJ Mira E Manes S. Extracellular Superoxide Dismutase, the Endothelial Basement Membrane, and the WNT Pathway: New Players in Vascular Normalization and Tumor Infiltration by T-Cells. Front Immunol (2020) 11:579552. doi: 10.3389/fimmu.2020.579552 33250894 PMC7673374

[17] Si W Shen J Zheng H Fan W. The Role and Mechanisms of Action of microRNAs in Cancer Drug Resistance. Clin Epigenet (2019) 11:25. doi: 10.1186/s13148-018-0587-8 PMC6371621 30744689

[18] Hossian AKMN Mackenzie GG Mattheolabakis G. Combination of Mir−143 and Mir−506 Reduces Lung and Pancreatic Cancer Cell Growth Through the Downregulation of Cyclin−Dependent Kinases. Oncol Rep (2021) 45:2. doi: 10.3892/or.2021.7953 33649787 PMC7876997

[19] Tahamtan A Teymoori-Rad M Nakstad B Salimi V. Anti-Inflammatory MicroRNAs and Their Potential for Inflammatory Diseases Treatment. Front Immunol (2018) 9:1377. doi: 10.3389/fimmu.2018.01377 29988529 PMC6026627

[20] Chung JY Thone MN Kwon YJ. COVID-19 Vaccines: The Status and Perspectives in Delivery Points of View. Adv Drug Delivery Rev (2020) 170:1–25. doi: 10.1016/j.addr.2020.12.011 PMC7759095 33359141

[21] Chung YH Beiss V Fiering SN Steinmetz NF. COVID-19 Vaccine Frontrunners and Their Nanotechnology Design. ACS Nano (2020) 14:12522–37. doi: 10.1021/acsnano.0c07197 33034449

[22] Pushparajah D Jimenez S Wong S Alattas H Nafissi N Slavcev RA. Advances in Gene-Based Vaccine Platforms to Address the COVID-19 Pandemic. Adv Drug Delivery Rev (2021) 170:113–41. doi: 10.1016/j.addr.2021.01.003 PMC7789827 33422546

[23] Labatut AE Mattheolabakis G. Non-Viral Based miR Delivery and Recent Developments. Eur J Pharm Biopharm (2018) 128:82–90. doi: 10.1016/j.ejpb.2018.04.018 29679644 PMC5984722

[24] Hossian A Mackenzie GG Mattheolabakis G. miRNAs in Gastrointestinal Diseases: Can We Effectively Deliver RNA-Based Therapeutics Orally? Nanomed (Lond) (2019) 14:2873–89. doi: 10.2217/nnm-2019-0180 PMC7026766 31735124

[25] Macha MA Seshacharyulu P Krishn SR Pai P Rachagani S Jain M. MicroRNAs (miRNAs) as Biomarker(s) for Prognosis and Diagnosis of Gastrointestinal (GI) Cancers. Curr Pharm Des (2014) 20:5287–97. doi: 10.2174/1381612820666140128213117 PMC4113605 24479799

[26] Mattheolabakis G Mikelis CM. Nanoparticle Delivery and Tumor Vascular Normalization: The Chicken or The Egg? Front Oncol (2019) 9:1227. doi: 10.3389/fonc.2019.01227 31799190 PMC6863425

[27] Bader AG Brown D Winkler M. The Promise of microRNA Replacement Therapy. Cancer Res (2010) 70:7027–30. doi: 10.1158/0008-5472.CAN-10-2010 PMC2940943 20807816

[28] Li L Yuan L Luo J Gao J Guo J Xie X. MiR-34a Inhibits Proliferation and Migration of Breast Cancer Through Down-Regulation of Bcl-2 and SIRT1. Clin Exp Med (2013) 13:109–17. doi: 10.1007/s10238-012-0186-5 22623155

[29] Wang JX Zhang QJ Pei SG Yang BL. Effect and Mechanism of miR-34a on Proliferation, Apoptosis and Invasion of Laryngeal Carcinoma Cells. Asian Pac J Trop Med (2016) 9:494–8. doi: 10.1016/j.apjtm.2016.03.018 27261861

[30] Zhang L Liao Y Tang L. MicroRNA-34 Family: A Potential Tumor Suppressor and Therapeutic Candidate in Cancer. J Exp Clin Cancer Res (2019) 38:53. doi: 10.1186/s13046-019-1059-5 30717802 PMC6360685

[31] Bonneau E Neveu B Kostantin E Tsongalis GJ De Guire V. How Close Are miRNAs From Clinical Practice? A Perspective on the Diagnostic and Therapeutic Market. EJIFCC (2019) 30:114–27. doi: 10.3389/fgene.2019.00478 PMC6599191 31263388

[32] Hanna J Hossain GS Kocerha J. The Potential for microRNA Therapeutics and Clinical Research. Front Genet (2019) 10:478. doi: 10.3389/fgene.2019.00478 31156715 PMC6532434

[33] Chakraborty C Sharma AR Sharma G Lee SS. Therapeutic Advances of miRNAs: A Preclinical and Clinical Update. J Adv Res (2021) 28:127–38. doi: 10.1016/j.jare.2020.08.012 PMC7753224 33364050

[34] Landskroner-Eiger S Moneke I Sessa WC. miRNAs as Modulators of Angiogenesis. Cold Spring Harb Perspect Med (2013) 3:a006643. doi: 10.1101/cshperspect.a006643 23169571 PMC3552340

[35] Peng Y Croce CM. The Role of MicroRNAs in Human Cancer. Signal Transduct Target Ther (2016) 1:15004. doi: 10.1038/sigtrans.2015.4 29263891 PMC5661652

[36] Fish JE Srivastava D. MicroRNAs: Opening a New Vein in Angiogenesis Research. Sci Signal (2009) 2:pe1. doi: 10.1126/scisignal.252pe1 19126861 PMC2680274

[37] Finn NA Searles CD. Intracellular and Extracellular miRNAs in Regulation of Angiogenesis Signaling. Curr Angiogenes (2012) 4:299–307. doi: 10.2174/2211552811201040299 23914347 PMC3729401

[38] Ma J Waxman DJ. Combination of Antiangiogenesis With Chemotherapy for More Effective Cancer Treatment. Mol Cancer Ther (2008) 7:3670–84. doi: 10.1158/1535-7163.MCT-08-0715 PMC2637411 19074844

[39] Senger DR Galli SJ Dvorak AM Perruzzi CA Harvey VS Dvorak HF. Tumor Cells Secrete a Vascular Permeability Factor That Promotes Accumulation of Ascites Fluid. Science (1983) 219:983–5. doi: 10.1126/science.6823562 6823562

[40] Leung DW Cachianes G Kuang WJ Goeddel DV Ferrara N. Vascular Endothelial Growth Factor Is a Secreted Angiogenic Mitogen. Science (1989) 246:1306–9. doi: 10.1126/science.2479986 2479986

[41] Carmeliet P Ferreira V Breier G Pollefeyt S Kieckens L Gertsenstein M. Abnormal Blood Vessel Development and Lethality in Embryos Lacking a Single VEGF Allele. Nature (1996) 380:435–9. doi: 10.1038/380435a0 8602241

[42] Ferrara N Carver-Moore K Chen H Dowd M Lu L O'shea KS. Heterozygous Embryonic Lethality Induced by Targeted Inactivation of the VEGF Gene. Nature (1996) 380:439–42. doi: 10.1038/380439a0 8602242

[43] Ferrara N Hillan KJ Novotny W. Bevacizumab (Avastin), a Humanized Anti-VEGF Monoclonal Antibody for Cancer Therapy. Biochem Biophys Res Commun (2005) 333:328–35. doi: 10.1016/j.bbrc.2005.05.132 15961063

[44] Ferrara N Kerbel RS. Angiogenesis as a Therapeutic Target. Nature (2005) 438:967–74. doi: 10.1038/nature04483 16355214

[45] Hurwitz H Fehrenbacher L Novotny W Cartwright T Hainsworth J Heim W. Bevacizumab Plus Irinotecan, Fluorouracil, and Leucovorin for Metastatic Colorectal Cancer. N Engl J Med (2004) 350:2335–42. doi: 10.1056/NEJMoa032691 15175435

[46] Herbst RS Heymach JV O'reilly MS Onn A Ryan AJ. Vandetanib (ZD6474): An Orally Available Receptor Tyrosine Kinase Inhibitor That Selectively Targets Pathways Critical for Tumor Growth and Angiogenesis. Expert Opin Investig Drugs (2007) 16:239–49. doi: 10.1517/13543784.16.2.239 17243944

[47] Houghton PJ. Everolimus. Clin Cancer Res (2010) 16:1368–72. doi: 10.1158/1078-0432.CCR-09-1314 PMC3003868 20179227

[48] Govindaraj C Madondo M Kong YY Tan P Wei A Plebanski M. Lenalidomide-Based Maintenance Therapy Reduces TNF Receptor 2 on CD4 T Cells and Enhances Immune Effector Function in Acute Myeloid Leukemia Patients. Am J Hematol (2014) 89:795–802. doi: 10.1002/ajh.23746 24757092

[49] Fink EC Ebert BL. The Novel Mechanism of Lenalidomide Activity. Blood (2015) 126:2366–9. doi: 10.1182/blood-2015-07-567958 PMC4653765 26438514

[50] Hsieh JJ Purdue MP Signoretti S Swanton C Albiges L Schmidinger M. Renal Cell Carcinoma. Nat Rev Dis Primers (2017) 3:17009. doi: 10.1038/nrdp.2017.9 28276433 PMC5936048

[51] Zhu YJ Zheng B Wang HY Chen L. New Knowledge of the Mechanisms of Sorafenib Resistance in Liver Cancer. Acta Pharmacol Sin (2017) 38:614–22. doi: 10.1038/aps.2017.5 PMC5457690 28344323

[52] Abou-Alfa GK Meyer T Cheng AL El-Khoueiry AB Rimassa L Ryoo BY. Cabozantinib in Patients With Advanced and Progressing Hepatocellular Carcinoma. N Engl J Med (2018) 379:54–63. doi: 10.1056/NEJMoa1717002 29972759 PMC7523244

[53] Nci. Angiogenesis Inhibitors (2018). Available at: https://www.cancer.gov/about-cancer/treatment/types/immunotherapy/angiogenesis-inhibitors-fact-sheet (Accessed 3.24 2021).

[54] Fondevila F Mendez-Blanco C Fernandez-Palanca P Gonzalez-Gallego J Mauriz JL. Anti-Tumoral Activity of Single and Combined Regorafenib Treatments in Preclinical Models of Liver and Gastrointestinal Cancers. Exp Mol Med (2019) 51:1–15. doi: 10.1038/s12276-019-0308-1 PMC6802659 31551425

[55] Ebos JM Lee CR Cruz-Munoz W Bjarnason GA Christensen JG Kerbel RS. Accelerated Metastasis After Short-Term Treatment With a Potent Inhibitor of Tumor Angiogenesis. Cancer Cell (2009) 15:232–9. doi: 10.1016/j.ccr.2009.01.021 PMC4540346 19249681

[56] Paez-Ribes M Allen E Hudock J Takeda T Okuyama H Vinals F. Antiangiogenic Therapy Elicits Malignant Progression of Tumors to Increased Local Invasion and Distant Metastasis. Cancer Cell (2009) 15:220–31. doi: 10.1016/j.ccr.2009.01.027 PMC2874829 19249680

[57] Wang D Tan C Xiao F Zou L Wang L Wei Y. The "Inherent Vice" in the Anti-Angiogenic Theory may Cause the Highly Metastatic Cancer to Spread More Aggressively. Sci Rep (2017) 7:2365. doi: 10.1038/s41598-017-02534-1 28539645 PMC5443774

[58] Lugano R Ramachandran M Dimberg A. Tumor Angiogenesis: Causes, Consequences, Challenges and Opportunities. Cell Mol Life Sci (2020) 77:1745–70. doi: 10.1007/s00018-019-03351-7 PMC7190605 31690961

[59] Rust R Gantner C Schwab ME. Pro- and Antiangiogenic Therapies: Current Status and Clinical Implications. FASEB J (2019) 33:34–48. doi: 10.1096/fj.201800640RR 30085886

[60] Scappaticci FA Skillings JR Holden SN Gerber HP Miller K Kabbinavar F. Arterial Thromboembolic Events in Patients With Metastatic Carcinoma Treated With Chemotherapy and Bevacizumab. J Natl Cancer Inst (2007) 99:1232–9. doi: 10.1093/jnci/djm086 17686822

[61] Chen HX Cleck JN. Adverse Effects of Anticancer Agents That Target the VEGF Pathway. Nat Rev Clin Oncol (2009) 6:465–77. doi: 10.1038/nrclinonc.2009.94 19581909

[62] Touyz RM Lang NN Herrmann J Van Den Meiracker AH Danser AHJ. Recent Advances in Hypertension and Cardiovascular Toxicities With Vascular Endothelial Growth Factor Inhibition. Hypertension (2017) 70:220–6. doi: 10.1161/HYPERTENSIONAHA.117.08856 PMC5509510 28630211

[63] Touyz RM Herrmann J. Cardiotoxicity With Vascular Endothelial Growth Factor Inhibitor Therapy. NPJ Precis Oncol (2018) 2:13. doi: 10.1038/s41698-018-0056-z 30202791 PMC5988734

[64] Seymour JF Pfreundschuh M Trneny M Sehn LH Catalano J Csinady E. R-CHOP With or Without Bevacizumab in Patients With Previously Untreated Diffuse Large B-Cell Lymphoma: Final MAIN Study Outcomes. Haematologica (2014) 99:1343–9. doi: 10.3324/haematol.2013.100818 PMC4116833 24895339

[65] Bailey CE Parikh AA. Assessment of the Risk of Antiangiogenic Agents Before and After Surgery. Cancer Treat Rev (2018) 68:38–46. doi: 10.1016/j.ctrv.2018.05.002 29793113

[66] Bodnar RJ. Anti-Angiogenic Drugs: Involvement in Cutaneous Side Effects and Wound-Healing Complication. Adv Wound Care (New Rochelle) (2014) 3:635–46. doi: 10.1089/wound.2013.0496 PMC4183909 25302138

[67] Davis-Dusenbery BN Hata A. MicroRNA in Cancer: The Involvement of Aberrant MicroRNA Biogenesis Regulatory Pathways. Genes Cancer (2010) 1:1100–14. doi: 10.1177/1947601910396213 PMC3083114 21533017

[68] Wang Y Wang L Chen C Chu X. New Insights Into the Regulatory Role of microRNA in Tumor Angiogenesis and Clinical Implications. Mol Cancer (2018) 17:22. doi: 10.1186/s12943-018-0766-4 29415727 PMC5804051

[69] Hu J Cheng Y Li Y Jin Z Pan Y Liu G. microRNA-128 Plays a Critical Role in Human Non-Small Cell Lung Cancer Tumourigenesis, Angiogenesis and Lymphangiogenesis by Directly Targeting Vascular Endothelial Growth Factor-C. Eur J Cancer (2014) 50:2336–50. doi: 10.1016/j.ejca.2014.06.005 25001183

[70] Yang L Dong C Yang J Yang L Chang N Qi C. MicroRNA-26b-5p Inhibits Mouse Liver Fibrogenesis and Angiogenesis by Targeting PDGF Receptor-Beta. Mol Ther Nucleic Acids (2019) 16:206–17. doi: 10.1016/j.omtn.2019.02.014 PMC6426711 30901579

[71] Sun T Yin L Kuang H. miR-181a/B-5p Regulates Human Umbilical Vein Endothelial Cell Angiogenesis by Targeting PDGFRA. Cell Biochem Funct (2020) 38:222–30. doi: 10.1002/cbf.3472 31879991

[72] Yamakuchi M Ferlito M Lowenstein CJ. miR-34a Repression of SIRT1 Regulates Apoptosis. Proc Natl Acad Sci USA (2008) 105:13421–6. doi: 10.1073/pnas.0801613105 PMC2533205 18755897

[73] Zhao T Li J Chen AF. MicroRNA-34a Induces Endothelial Progenitor Cell Senescence and Impedes Its Angiogenesis via Suppressing Silent Information Regulator 1. Am J Physiol Endocrinol Metab (2010) 299:E110–116. doi: 10.1152/ajpendo.00192.2010 PMC2904051 20424141

[74] Tabuchi T Satoh M Itoh T Nakamura M. MicroRNA-34a Regulates the Longevity-Associated Protein SIRT1 in Coronary Artery Disease: Effect of Statins on SIRT1 and microRNA-34a Expression. Clin Sci (Lond) (2012) 123:161–71. doi: 10.1042/CS20110563 22364258

[75] Shi S Jin Y Song H Chen X. MicroRNA-34a Attenuates VEGF-Mediated Retinal Angiogenesis via Targeting Notch1. Biochem Cell Biol (2019) 97:423–30. doi: 10.1139/bcb-2018-0304 30571142

[76] Yu G Yao W Xiao W Li H Xu H Lang B. MicroRNA-34a Functions as an Anti-Metastatic microRNA and Suppresses Angiogenesis in Bladder Cancer by Directly Targeting CD44. J Exp Clin Cancer Res (2014) 33:779. doi: 10.1186/s13046-014-0115-4 25551284 PMC4311467

[77] Su G Sun G Liu H Shu L Liang Z. Downregulation of miR-34a Promotes Endothelial Cell Growth and Suppresses Apoptosis in Atherosclerosis by Regulating Bcl-2. Heart Vessels (2018) 33:1185–94. doi: 10.1007/s00380-018-1169-6 29704100

[78] Vinall RL Ripoll AZ Wang S Pan CX Devere White RW. MiR-34a Chemosensitizes Bladder Cancer Cells to Cisplatin Treatment Regardless of P53-Rb Pathway Status. Int J Cancer (2012) 130:2526–38. doi: 10.1002/ijc.26256 PMC4568996 21702042

[79] Song C Lu P Sun G Yang L Wang Z Wang Z. miR-34a Sensitizes Lung Cancer Cells to Cisplatin via P53/miR-34a/MYCN Axis. Biochem Biophys Res Commun (2017) 482:22–7. doi: 10.1016/j.bbrc.2016.11.037 27836543

[80] Zheng SZ Sun P Wang JP Liu Y Gong W Liu J. MiR-34a Overexpression Enhances the Inhibitory Effect of Doxorubicin on HepG2 Cells. World J Gastroenterol (2019) 25:2752–62. doi: 10.3748/wjg.v25.i22.2752 PMC6580351 31235998

[81] Jian C Tu MJ Ho PY Duan Z Zhang Q Qiu JX. Co-Targeting of DNA, RNA, and Protein Molecules Provides Optimal Outcomes for Treating Osteosarcoma and Pulmonary Metastasis in Spontaneous and Experimental Metastasis Mouse Models. Oncotarget (2017) 8:30742–55. doi: 10.18632/oncotarget.16372 PMC5458164 28415566

[82] Wen D Peng Y Lin F Singh RK Mahato RI. Micellar Delivery of miR-34a Modulator Rubone and Paclitaxel in Resistant Prostate Cancer. Cancer Res (2017) 77:3244–54. doi: 10.1158/0008-5472.CAN-16-2355 PMC5673080 28428276

[83] Fang JH Zhou HC Zeng C Yang J Liu Y Huang X. MicroRNA-29b Suppresses Tumor Angiogenesis, Invasion, and Metastasis by Regulating Matrix Metalloproteinase 2 Expression. Hepatology (2011) 54:1729–40. doi: 10.1002/hep.24577 21793034

[84] Kriegel AJ Liu Y Fang Y Ding X Liang M. The miR-29 Family: Genomics, Cell Biology, and Relevance to Renal and Cardiovascular Injury. Physiol Genomics (2012) 44:237–44. doi: 10.1152/physiolgenomics.00141.2011 PMC3289120 22214600

[85] Chen HX Xu XX Tan BZ Zhang Z Zhou XD. MicroRNA-29b Inhibits Angiogenesis by Targeting VEGFA Through the MAPK/ERK and PI3K/Akt Signaling Pathways in Endometrial Carcinoma. Cell Physiol Biochem (2017) 41:933–46. doi: 10.1159/000460510 28222438

[86] Li Y Cai B Shen L Dong Y Lu Q Sun S. MiRNA-29b Suppresses Tumor Growth Through Simultaneously Inhibiting Angiogenesis and Tumorigenesis by Targeting Akt3. Cancer Lett (2017) 397:111–9. doi: 10.1016/j.canlet.2017.03.032 28365400

[87] Zhang H Bai M Deng T Liu R Wang X Qu Y. Cell-Derived Microvesicles Mediate the Delivery of miR-29a/C to Suppress Angiogenesis in Gastric Carcinoma. Cancer Lett (2016) 375:331–9. doi: 10.1016/j.canlet.2016.03.026 27000664

[88] Sun DM Tang BF Li ZX Guo HB Cheng JL Song PP. MiR-29c Reduces the Cisplatin Resistance of Non-Small Cell Lung Cancer Cells by Negatively Regulating the PI3K/Akt Pathway. Sci Rep (2018) 8:8007. doi: 10.1038/s41598-018-26381-w 29789623 PMC5964122

[89] Huang L Hu C Chao H Wang R Lu H Li H. miR-29c Regulates Resistance to Paclitaxel in Nasopharyngeal Cancer by Targeting ITGB1. Exp Cell Res (2019) 378:1–10. doi: 10.1016/j.yexcr.2019.02.012 30779921

[90] Shen H Li L Yang S Wang D Zhong S Zhao J. MicroRNA-29a Contributes to Drug-Resistance of Breast Cancer Cells to Adriamycin Through PTEN/AKT/GSK3beta Signaling Pathway. Gene (2016) 593:84–90. doi: 10.1016/j.gene.2016.08.016 27523474

[91] Shi X Valizadeh A Mir SM Asemi Z Karimian A Majidina M. miRNA-29a Reverses P-Glycoprotein-Mediated Drug Resistance and Inhibits Proliferation via Up-Regulation of PTEN in Colon Cancer Cells. Eur J Pharmacol (2020) 880:173138. doi: 10.1016/j.ejphar.2020.173138 32416187

[92] Li Y Zhang Z Xiao Z Lin Y Luo T Zhou Q. Chemotherapy-Mediated miR-29b Expression Inhibits the Invasion and Angiogenesis of Cervical Cancer. Oncotarget (2017) 8:14655–65. doi: 10.18632/oncotarget.14738 PMC5362433 28122338

[93] Poliseno L Tuccoli A Mariani L Evangelista M Citti L Woods K. MicroRNAs Modulate the Angiogenic Properties of HUVECs. Blood (2006) 108:3068–71. doi: 10.1182/blood-2006-01-012369 16849646

[94] Bartel DP. MicroRNAs: Target Recognition and Regulatory Functions. Cell (2009) 136:215–33. doi: 10.1016/j.cell.2009.01.002 PMC3794896 19167326

[95] Nicoli S Knyphausen CP Zhu LJ Lakshmanan A Lawson ND. miR-221 Is Required for Endothelial Tip Cell Behaviors During Vascular Development. Dev Cell (2012) 22:418–29. doi: 10.1016/j.devcel.2012.01.008 PMC3285411 22340502

[96] Chen Y Banda M Speyer CL Smith JS Rabson AB Gorski DH. Regulation of the Expression and Activity of the Antiangiogenic Homeobox Gene GAX/MEOX2 by ZEB2 and microRNA-221. Mol Cell Biol (2010) 30:3902–13. doi: 10.1128/MCB.01237-09 PMC2916411 20516212

[97] Wu YH Hu TF Chen YC Tsai YN Tsai YH Cheng CC. The Manipulation of miRNA-Gene Regulatory Networks by KSHV Induces Endothelial Cell Motility. Blood (2011) 118:2896–905. doi: 10.1182/blood-2011-01-330589 21715310

[98] Li Y Yan C Fan J Hou Z Han Y. MiR-221-3p Targets Hif-1alpha to Inhibit Angiogenesis in Heart Failure. Lab Invest (2021) 101:104–15. doi: 10.1038/s41374-020-0450-3 32873879

[99] Mercatelli N Coppola V Bonci D Miele F Costantini A Guadagnoli M. The Inhibition of the Highly Expressed miR-221 and miR-222 Impairs the Growth of Prostate Carcinoma Xenografts in Mice. PloS One (2008) 3:e4029. doi: 10.1371/journal.pone.0004029 19107213 PMC2603596

[100] Le Sage C Nagel R Egan DA Schrier M Mesman E Mangiola A. Regulation of the P27(Kip1) Tumor Suppressor by miR-221 and miR-222 Promotes Cancer Cell Proliferation. EMBO J (2007) 26:3699–708. doi: 10.1038/sj.emboj.7601790 PMC1949005 17627278

[101] Miller TE Ghoshal K Ramaswamy B Roy S Datta J Shapiro CL. MicroRNA-221/222 Confers Tamoxifen Resistance in Breast Cancer by Targeting p27Kip1. J Biol Chem (2008) 283:29897–903. doi: 10.1074/jbc.M804612200 PMC2573063 18708351

[102] Garofalo M Quintavalle C Di Leva G Zanca C Romano G Taccioli C. MicroRNA Signatures of TRAIL Resistance in Human Non-Small Cell Lung Cancer. Oncogene (2008) 27:3845–55. doi: 10.1038/onc.2008.6 18246122

[103] Amini-Farsani Z Sangtarash MH Shamsara M Teimori H. MiR-221/222 Promote Chemoresistance to Cisplatin in Ovarian Cancer Cells by Targeting PTEN/PI3K/AKT Signaling Pathway. Cytotechnology (2018) 70:203–13. doi: 10.1007/s10616-017-0134-z PMC5809651 28887606

[104] Li S Li Q Lu J Zhao Q Li D Shen L. Targeted Inhibition of miR-221/222 Promotes Cell Sensitivity to Cisplatin in Triple-Negative Breast Cancer MDA-MB-231 Cells. Front Genet (2019) 10:1278. doi: 10.3389/fgene.2019.01278 32010177 PMC6971202

[105] Chen D Yan W Liu Z Zhang Z Zhu L Liu W. Downregulation of miR-221 Enhances the Sensitivity of Human Oral Squamous Cell Carcinoma Cells to Adriamycin Through Upregulation of TIMP3 Expression. BioMed Pharmacother (2016) 77:72–8. doi: 10.1016/j.biopha.2015.12.002 26796268

[106] Du L Ma S Wen X Chai J Zhou D. Oral Squamous Cell Carcinoma Cells Are Resistant to Doxorubicin Through Upregulation of Mir221. Mol Med Rep (2017) 16:2659–67. doi: 10.3892/mmr.2017.6915 PMC5547975 28677788

[107] Wang Y Zhao Y Herbst A Kalinski T Qin J Wang X. miR-221 Mediates Chemoresistance of Esophageal Adenocarcinoma by Direct Targeting of DKK2 Expression. Ann Surg (2016) 264:804–14. doi: 10.1097/SLA.0000000000001928 27501171

[108] Ni L Xu J Zhao F Dai X Tao J Pan J. MiR-221-3p-Mediated Downregulation of MDM2 Reverses the Paclitaxel Resistance of Non-Small Cell Lung Cancer In Vitro and In Vivo. Eur J Pharmacol (2021) 899:174054. doi: 10.1016/j.ejphar.2021.174054 33771522

[109] Lei Z Li B Yang Z Fang H Zhang GM Feng ZH. Regulation of HIF-1alpha and VEGF by miR-20b Tunes Tumor Cells to Adapt to the Alteration of Oxygen Concentration. PloS One (2009) 4:e7629. doi: 10.1371/journal.pone.0007629 19893619 PMC2764090

[110] Chou J Lin JH Brenot A Kim JW Provot S Werb Z. GATA3 Suppresses Metastasis and Modulates the Tumour Microenvironment by Regulating microRNA-29b Expression. Nat Cell Biol (2013) 15:201–13. doi: 10.1038/ncb2672 PMC3660859 23354167

[111] Long J Wang Y Wang W Chang BH Danesh FR. Identification of microRNA-93 as a Novel Regulator of Vascular Endothelial Growth Factor in Hyperglycemic Conditions. J Biol Chem (2010) 285:23457–65. doi: 10.1074/jbc.M110.136168 PMC2906336 20501654

[112] Li F Liang X Chen Y Li S Liu J. Role of microRNA-93 in Regulation of Angiogenesis. Tumour Biol (2014) 35:10609–13. doi: 10.1007/s13277-014-2605-6 25217985

[113] Tirpe A Gulei D Tirpe GR Nutu A Irimie A Campomenosi P. Beyond Conventional: The New Horizon of Anti-Angiogenic microRNAs in Non-Small Cell Lung Cancer Therapy. Int J Mol Sci (2020) 21:8002. doi: 10.3390/ijms21218002 PMC7663714 33121202

[114] Chen Q Chen S Zhao J Zhou Y Xu L. MicroRNA-126: A New and Promising Player in Lung Cancer. Oncol Lett (2021) 21:35. doi: 10.3892/ol.2020.12296 33262827 PMC7693477

[115] Ye J Wu X Wu D Wu P Ni C Zhang Z. miRNA-27b Targets Vascular Endothelial Growth Factor C to Inhibit Tumor Progression and Angiogenesis in Colorectal Cancer. PloS One (2013) 8:e60687. doi: 10.1371/journal.pone.0060687 23593282 PMC3625233

[116] Liu HT Xing AY Chen X Ma RR Wang YW Shi DB. MicroRNA-27b, microRNA-101 and microRNA-128 Inhibit Angiogenesis by Down-Regulating Vascular Endothelial Growth Factor C Expression in Gastric Cancers. Oncotarget (2015) 6:37458–70. doi: 10.18632/oncotarget.6059 PMC4741941 26460960

[117] Muramatsu F Kidoya H Naito H Sakimoto S Takakura N. microRNA-125b Inhibits Tube Formation of Blood Vessels Through Translational Suppression of VE-Cadherin. Oncogene (2013) 32:414–21. doi: 10.1038/onc.2012.68 22391569

[118] Wu SY Rupaimoole R Shen F Pradeep S Pecot CV Ivan C. A miR-192-EGR1-HOXB9 Regulatory Network Controls the Angiogenic Switch in Cancer. Nat Commun (2016) 7:11169. doi: 10.1038/ncomms11169 27041221 PMC4822037

[119] Pecot CV Rupaimoole R Yang D Akbani R Ivan C Lu C. Tumour Angiogenesis Regulation by the miR-200 Family. Nat Commun (2013) 4:2427. doi: 10.1038/ncomms3427 24018975 PMC3904438

[120] Sun G Liu M Han H. Overexpression of microRNA-190 Inhibits Migration, Invasion, Epithelial-Mesenchymal Transition, and Angiogenesis Through Suppression of Protein Kinase B-Extracellular Signal-Regulated Kinase Signaling Pathway via Binding to Stanniocalicin 2 in Breast Cancer. J Cell Physiol (2019) 234:17824–38. doi: 10.1002/jcp.28409 30993707

[121] Chen QY Jiao DM Wu YQ Chen J Wang J Tang XL. MiR-206 Inhibits HGF-Induced Epithelial-Mesenchymal Transition and Angiogenesis in Non-Small Cell Lung Cancer via C-Met /PI3k/Akt/mTOR Pathway. Oncotarget (2016) 7:18247–61. doi: 10.18632/oncotarget.7570 PMC4951285 26919096

[122] Zhou Y Li S Li J Wang D Li Q. Effect of microRNA-135a on Cell Proliferation, Migration, Invasion, Apoptosis and Tumor Angiogenesis Through the IGF-1/PI3K/Akt Signaling Pathway in Non-Small Cell Lung Cancer. Cell Physiol Biochem (2017) 42:1431–46. doi: 10.1159/000479207 28715819

[123] Hossian A Sajib MS Tullar PE Mikelis CM Mattheolabakis G. Multipronged Activity of Combinatorial miR-143 and miR-506 Inhibits Lung Cancer Cell Cycle Progression and Angiogenesis. Vitro Sci Rep (2018) 8:10495. doi: 10.1038/s41598-018-28872-2 30002440 PMC6043488

[124] Belcheva A Wey JS Fan F Ellis LM. Expression of Vascular Endothelial Growth Factor Receptors (VEGF-Rs) on Human Breast Cancer Cells Confers Chemoresistance. Cancer Res (2004) 64:1000.14871831

[125] Stanton MJ Dutta S Zhang H Polavaram NS Leontovich AA Honscheid P. Autophagy Control by the VEGF-C/NRP-2 Axis in Cancer and Its Implication for Treatment Resistance. Cancer Res (2013) 73:160–71. doi: 10.1158/0008-5472.CAN-11-3635 PMC3805049 23149913

[126] Wang CA Harrell JC Iwanaga R Jedlicka P Ford HL. Vascular Endothelial Growth Factor C Promotes Breast Cancer Progression via a Novel Antioxidant Mechanism That Involves Regulation of Superoxide Dismutase 3. Breast Cancer Res (2014) 16:462. doi: 10.1186/s13058-014-0462-2 25358638 PMC4303136

[127] Zhu X Li H Long L Hui L Chen H Wang X. miR-126 Enhances the Sensitivity of Non-Small Cell Lung Cancer Cells to Anticancer Agents by Targeting Vascular Endothelial Growth Factor A. Acta Biochim Biophys Sin (Shanghai) (2012) 44:519–26. doi: 10.1093/abbs/gms026 22510476

[128] Zhao Y Tu MJ Yu YF Wang WP Chen QX Qiu JX. Combination Therapy With Bioengineered miR-34a Prodrug and Doxorubicin Synergistically Suppresses Osteosarcoma Growth. Biochem Pharmacol (2015) 98:602–13. doi: 10.1016/j.bcp.2015.10.015 PMC4725324 26518752

[129] Yu J Zhao Y Liu C Hu B Zhao M Ma Y. Synergistic Anti-Tumor Effect of Paclitaxel and miR-34a Combined With Ultrasound Microbubbles on Cervical Cancer In Vivo and In Vitro. Clin Transl Oncol (2020) 22:60–9. doi: 10.1007/s12094-019-02131-w 31093891

[130] Shi S Han L Deng L Zhang Y Shen H Gong T. Dual Drugs (microRNA-34a and Paclitaxel)-Loaded Functional Solid Lipid Nanoparticles for Synergistic Cancer Cell Suppression. J Control Release (2014) 194:228–37. doi: 10.1016/j.jconrel.2014.09.005 25220161

[131] Soltani-Sedeh H Irani S Mirfakhraie R Soleimani M. Potential Using of microRNA-34A in Combination With Paclitaxel in Colorectal Cancer Cells. J Cancer Res Ther (2019) 15:32–7. doi: 10.4103/jcrt.JCRT_267_17 30880751

[132] Zhang L Yang X Lv Y Xin X Qin C Han X. Cytosolic Co-Delivery of miRNA-34a and Docetaxel With Core-Shell Nanocarriers via Caveolae-Mediated Pathway for the Treatment of Metastatic Breast Cancer. Sci Rep (2017) 7:46186. doi: 10.1038/srep46186 28383524 PMC5382875

[133] Zhang Q Wang J Li N Liu Z Chen Z Li Z. miR-34a Increases the Sensitivity of Colorectal Cancer Cells to 5-Fluorouracil In Vitro and In Vivo. Am J Cancer Res (2018) 8:280–90. PMC5835695 29511598

[134] Xu J Zhang G Luo X Wang D Zhou W Zhang Y. Co-Delivery of 5-Fluorouracil and miRNA-34a Mimics by Host-Guest Self-Assembly Nanocarriers for Efficacious Targeted Therapy in Colorectal Cancer Patient-Derived Tumor Xenografts. Theranostics (2021) 11:2475–89. doi: 10.7150/thno.52076 PMC7797688 33500737

[135] Jilek JL Tu MJ Zhang C Yu AM. Pharmacokinetic and Pharmacodynamic Factors Contribute to Synergism Between Let-7c-5p and 5-Fluorouracil in Inhibiting Hepatocellular Carcinoma Cell Viability. Drug Metab Dispos (2020) 48:1257–63. doi: 10.1124/dmd.120.000207 PMC7684025 33051247

[136] Costa PM Cardoso AL Custodia C Cunha P Pereira De Almeida L Pedroso De Lima MC. MiRNA-21 Silencing Mediated by Tumor-Targeted Nanoparticles Combined With Sunitinib: A New Multimodal Gene Therapy Approach for Glioblastoma. J Control Release (2015) 207:31–9. doi: 10.1016/j.jconrel.2015.04.002 25861727

[137] Passadouro M Pedroso De Lima MC Faneca H. MicroRNA Modulation Combined With Sunitinib as a Novel Therapeutic Strategy for Pancreatic Cancer. Int J Nanomed (2014) 9:3203–17. doi: 10.2147/IJN.S64456 PMC4086670 25061297

[138] Liu H Liu Z Jiang B Huo L Liu J Lu J. Synthetic miR-145 Mimic Enhances the Cytotoxic Effect of the Antiangiogenic Drug Sunitinib in Glioblastoma. Cell Biochem Biophys (2015) 72:551–7. doi: 10.1007/s12013-014-0501-8 25564360

[139] Kim SJ Oh JS Shin JY Lee KD Sung KW Nam SJ. Development of microRNA-145 for Therapeutic Application in Breast Cancer. J Control Release (2011) 155:427–34. doi: 10.1016/j.jconrel.2011.06.026 21723890

[140] Mittal A Chitkara D Behrman SW Mahato RI. Efficacy of Gemcitabine Conjugated and miRNA-205 Complexed Micelles for Treatment of Advanced Pancreatic Cancer. Biomaterials (2014) 35:7077–87. doi: 10.1016/j.biomaterials.2014.04.053 24836307

[141] Karaayvaz M Zhai H Ju J. miR-129 Promotes Apoptosis and Enhances Chemosensitivity to 5-Fluorouracil in Colorectal Cancer. Cell Death Dis (2013) 4:e659. doi: 10.1038/cddis.2013.193 23744359 PMC3702282

[142] Liu L Zheng W Song Y Du X Tang Y Nie J. miRNA-497 Enhances the Sensitivity of Colorectal Cancer Cells to Neoadjuvant Chemotherapeutic Drug. Curr Protein Pept Sci (2015) 16:310–5. doi: 10.2174/138920371604150429154142 25929865

[143] Zhang G Tian X Li Y Wang Z Li X Zhu C. miR-27b and miR-34a Enhance Docetaxel Sensitivity of Prostate Cancer Cells Through Inhibiting Epithelial-to-Mesenchymal Transition by Targeting ZEB1. BioMed Pharmacother (2018) 97:736–44. doi: 10.1016/j.biopha.2017.10.163 29102917

[144] Hu H Wang Z Tan C Liu X Zhang H Li K. Dihydroartemisinin/miR-29b Combination Therapy Increases the Pro-Apoptotic Effect of Dihydroartemisinin on Cholangiocarcinoma Cell Lines by Regulating Mcl-1 Expression. Adv Clin Exp Med (2020) 29:911–9. doi: 10.17219/acem/121919 32790250

[145] Lee J Choi KJ Moon SU Kim S. Theragnosis-Based Combined Cancer Therapy Using Doxorubicin-Conjugated microRNA-221 Molecular Beacon. Biomaterials (2016) 74:109–18. doi: 10.1016/j.biomaterials.2015.09.036 26454049

[146] Zhang Y He Y Lu LL Zhou ZY Wan NB Li GP. miRNA-192-5p Impacts the Sensitivity of Breast Cancer Cells to Doxorubicin via Targeting Peptidylprolyl Isomerase A. Kaohsiung J Med Sci (2019) 35:17–23. doi: 10.1002/kjm2.12004 30844143 PMC11900784

[147] Fu H Zhang J Pan T Ai S Tang L Wang F. Mir378a Enhances the Sensitivity of Liver Cancer to Sorafenib by Targeting VEGFR, PDGFRbeta and Craf. Mol Med Rep (2018) 17:4581–8. doi: 10.3892/mmr.2018.8390 29328399

[148] Xu H Zhao L Fang Q Sun J Zhang S Zhan C. MiR-338-3p Inhibits Hepatocarcinoma Cells and Sensitizes These Cells to Sorafenib by Targeting Hypoxia-Induced Factor 1alpha. PloS One (2014) 9:e115565. doi: 10.1371/journal.pone.0115565 25531114 PMC4274118

[149] Hejazi M Baghbani E Amini M Rezaei T Aghanejad A Mosafer J. MicroRNA-193a and Taxol Combination: A New Strategy for Treatment of Colorectal Cancer. J Cell Biochem (2020) 121:1388–99. doi: 10.1002/jcb.29374 31512793

[150] Esfandyari YB Doustvandi MA Amini M Baradaran B Zaer SJ Mozammel N. MicroRNA-143 Sensitizes Cervical Cancer Cells to Cisplatin: A Promising Anticancer Combination Therapy. Reprod Sci (2021) 28:2036–49. doi: 10.1007/s43032-021-00479-5 33569751

[151] Yu PN Yan MD Lai HC Huang RL Chou YC Lin WC. Downregulation of miR-29 Contributes to Cisplatin Resistance of Ovarian Cancer Cells. Int J Cancer (2014) 134:542–51. doi: 10.1002/ijc.28399 23904094

[152] Gajda E Godlewska M Mariak Z Nazaruk E Gawel D. Combinatory Treatment With miR-7-5p and Drug-Loaded Cubosomes Effectively Impairs Cancer Cells. Int J Mol Sci (2020) 21:5039. doi: 10.3390/ijms21145039 PMC7404280 32708846

[153] Sun Y Wu J Dong X Zhang J Meng C Liu G. MicroRNA-506-3p Increases the Response to PARP Inhibitors and Cisplatin by Targeting EZH2/beta-Catenin in Serous Ovarian Cancers. Transl Oncol (2021) 14:100987. doi: 10.1016/j.tranon.2020.100987 33360300 PMC7770486

[154] Golubovskaya VM Sumbler B Ho B Yemma M Cance WG. MiR-138 and MiR-135 Directly Target Focal Adhesion Kinase, Inhibit Cell Invasion, and Increase Sensitivity to Chemotherapy in Cancer Cells. Anticancer Agents Med Chem (2014) 14:18–28. doi: 10.2174/187152061401140108113435 23438844 PMC3883917

[155] Wang F Meng F Wong SCC Cho WCS Yang S Chan LWC. Combination Therapy of Gefitinib and miR-30a-5p may Overcome Acquired Drug Resistance Through Regulating the PI3K/AKT Pathway in Non-Small Cell Lung Cancer. Ther Adv Respir Dis (2020) 14:1753466620915156. doi: 10.1177/1753466620915156 32552611 PMC7303773

[156] Liang G Zhu Y Ali DJ Tian T Xu H Si K. Engineered Exosomes for Targeted Co-Delivery of miR-21 Inhibitor and Chemotherapeutics to Reverse Drug Resistance in Colon Cancer. J Nanobiotechnol (2020) 18:10. doi: 10.1186/s12951-019-0563-2 PMC6950820 31918721

[157] Xu X Chen X Xu M Liu X Pan B Qin J. miR-375-3p Suppresses Tumorigenesis and Partially Reverses Chemoresistance by Targeting YAP1 and SP1 in Colorectal Cancer Cells. Aging (Albany NY) (2019) 11:7357–85. doi: 10.18632/aging.102214 PMC6781994 31543507

[158] Xu F Ye ML Zhang YP Li WJ Li MT Wang HZ. MicroRNA-375-3p Enhances Chemosensitivity to 5-Fluorouracil by Targeting Thymidylate Synthase in Colorectal Cancer. Cancer Sci (2020) 111:1528–41. doi: 10.1111/cas.14356 PMC7226198 32073706

[159] Lai X Eberhardt M Schmitz U Vera J. Systems Biology-Based Investigation of Cooperating microRNAs as Monotherapy or Adjuvant Therapy in Cancer. Nucleic Acids Res (2019) 47:7753–66. doi: 10.1093/nar/gkz638 PMC6735922 31340025

[160] Rupaimoole R Han HD Lopez-Berestein G Sood AK. MicroRNA Therapeutics: Principles, Expectations, and Challenges. Chin J Cancer (2011) 30:368–70. doi: 10.5732/cjc.011.10186 PMC4013410 21627858

